# Assessment of soybean productivity and its changing factors in Japan based on the production cost statistics

**DOI:** 10.1016/j.heliyon.2024.e38396

**Published:** 2024-10-09

**Authors:** Sohei Kobayashi, Yoji Kunimitsu

**Affiliations:** a Division of Livestock Research, Kyushu Okinawa Agricultural Research Center, National Agriculture and Food Research Organization (NARO), Suya 2421, Koshi, Kumamoto, 861–1192, Japan; b Department of Research Promotion, Institute for Rural Development, NARO, Kannondai 2–1–6, Tsukuba, Ibaraki, 305–8609, Japan

**Keywords:** Land size, Soybean (*Glycine max* [L.] Merr.), Technological progress, Total factor productivity, Yield

## Abstract

Soybean (*Glycine max* [L.] Merr.) yields have remained below 2.0 t ha^−1^ for over 30 years in Asian countries, indicating long-term stagnation in productivity and production technology. This study aimed to measure the total factor productivity (TFP) of soybean production in Japan and to assess the factors affecting TFP and yield. The Färe–Primont index of TFP was measured for two agroclimatic regions using soybean production cost statistics from 1987 to 2015 to analyze the regression of TFP and yield with per-farm land size, time trends, and production-related input variables. The TFP increased by 1.5–2.4 % annually in the two regions, despite an annual decrease of 0.7 % in country's average yield. The TFP in the subarctic Hokkaido region improved by 9–15 % due to the land-size increase and progress of time, i.e., technological progress, during the period. While in the temperate main-island region, a 14 % TFP increase induced by increased land size was largely offset by the time–progress effect (−11 %), i.e., technological retrogression. Similar time–progress effects observed on yield indicated that technological changes occurred, with respect to cultivation methods. The TFP–improving land-size increase adversely resulted in a 9 % yield decrease in the main-island region accounting for 85 % of soybean cultivation area in Japan. The results further showed that TFP improved by saving production inputs, such as fertilizer, per unit area in both regions, and the yield rose with increased inputs in seed, agrochemicals, and rental services, depending on the region. This study revealed continual soybean TFP improvements across Japan due to land-size increases and input saving in addition to the regionally biased progress in cultivation technologies. The implications of the results are discussed from the perspective of improving TFP and yield while increasing the per-farm land size.

## 1 Introduction

Soybeans (*Glycine max* [L.] Merr.) are an essential legume crop that is processed for food, oil, and animal feed. Asian countries, such as Japan and China, have a long history of soybean cultivation for producing traditional foods (e.g., *Tofu*, *Miso*); however, the yield has remained below 2.0 t ha^−1^ over the last 30 years (Appendix 1; [[1], [2], [3]]). Katsura et al. [2] simulated and estimated the soybean yield potential in Japan to be 4–6 t ha^−1^ depending on the location, displaying that the low yield was not due to a limited yield potential. The factors responsible for low yields in Japan have been intensively studied in the field and laboratory for over 20 years [[2], [3], [4], [5], [6], [7], [8]]. Several countermeasure technologies were developed, e.g., locally adapted tillage methods and high–yield cultivars, some of which were disseminated to farms in subsidized projects [[9], [10], [11]]. Despite these efforts, no apparent increase in yield was observed, indicating a long-term stagnation in productivity and production technology. Similar results have been reported for China and India (Appendix 1) [12,13]. Therefore, there is a need to reassess the factors responsible for low soybean yields and to improve the understanding of technological changes in soybean production. Japan, where yield stagnation started earlier than in other Asian countries, is a good study object for such an assessment and understanding.

The yield displays the productivity standardized by land area (i.e., land productivity), whereas the total factor productivity (TFP) measured as the ratio of aggregate outputs (e.g., total soybean yield in a farm) to aggregate inputs (e.g., fertilizer, machinery, and land used for soybean production on the farm) shows the overall productivity standardized by the multiple inputs based on economic theories of production. Although both values serve as indicators of productivity and technological level, the latter TFP is advantageous for long-term assessments as it considers the varying quantities of production inputs between years. Moreover, the latter TFP can express, as an increase in value, that the improvement in productivity is realized by reducing the inputs to a greater extent than the outputs. Based on the soybean production cost statistics of Japan for the last 40 years, Kobayashi and Kunimitsu [14,15] measured soybean TFP using the multilateral Törnqvist index corresponding to the trans–log production function and estimated that the TFP increased annually at 0.7–1.0 %, despite the yield stagnation. In China, where soybean yield has also stagnated, Liu and Li [16] and Si and Wang [17] reported the annual soybean TFP growth of 0.42 % and 1.5 % between the 1980s and 2000s by estimating the Cobb–Douglas and stochastic frontier production functions, respectively, using province–level cost statistics. In the United State of America and Argentina, several studies have analyzed the intra– and inter–state yield differences or the potential–actual yield gap, by using on-station and -farm trials as well as statistical and yield-contest data, and proposed technological solutions for further increasing the yield [[18], [19], [20], [21], [22]]. However, very few studies have measured soybean TFP, likely owing to a steadily increasing yield being assumed to indicate progress in productivity and production technology.

The soybean–cultivating land area per soybean–producing farm (hereafter referred to as the land size) increased by 11 times from 1987 to 2015 in Japan due to the outflow and aging of the rural population (Appendix 2; [23]) and has likely also increased in other Asian countries (Appendix 3; [1,24]). The drastic increase in the land size, accompanied by field consolidation and machinery upsizing, is considered to have improved soybean TFP by the economies of scale. However, such a land-size expansion could also lead to extensive production management with decreases in some inputs per unit area, likely owing to the fixed or limited labor at the farm level, and consequently to reducing the yield per unit area (Appendix 4) [15]. Thus, the land size expansion was assumed as a major factor responsible for both the previously reported soybean TFP improvement and yield stagnation in Japan, although it is necessary to assess and verify the multiple effects of the expansion on TFP and yield. The drastic land-size increase is further considered to have conflicted with the farmers’ adoption of land-size–irrelevant production technologies that typically need additional inputs to stabilize or increase the yield, such as those developed and recommended by public research organizations (e.g., new cultivars, early sowing, manure application, and precise but expensive machinery). Thus, it is also necessary to assess whether the progress of the technologies (i.e., land-size–independent TFP and yield growth) occurred or not, and what production–input elements affected TFP and yield independently of land size. Such assessments would assist policymakers and scientists to precisely understand how soybean TFP rose and yield stagnated or declined with increasing land size, and to identify promising input elements and research targets for improving TFP and yield while increasing land size. However, an assessment that analyzes both soybean TFP and yield, while considering land size, has not been conducted previously in Japan and other countries.

The objectives of this study were to measure soybean TFP in two agroclimatic regions comprising the land of Japan and to assess the effects of land size and other production inputs on TFP and yield in each region using regression analyses. To achieve these objectives, this study used soybean production cost statistics that had been compiled annually by the Government of Japan for multiple land-size sections in the two regions with the data of unit-area yield between 1987 and 2015 [23]. The statistics involving different land-size sections and yield data were suitable and advantageous for the current assessment as compared to state–level summary statistics missing land-size and yield data in other countries [[15], [16], [17],^25^]. The TFP was measured as the Färe–Primont index by data envelope analysis (DEA), as this method can calculate TFP values with ideal multiplicativity and transitivity characteristics based on a limited amount of statistical data without assuming and estimating a specific production function [[26], [27], [28], [29]], which is in contrast to other methods, such as those with the Malmquist index [30,31] and stochastic frontier analysis (SFA) [17,32]. The Färe–Primont soybean TFP and yield were regressed against land size, time trend (year), and other production input variables to estimate their contributions to the TFP and yield variation during 1987–2015. To the best of our knowledge, few studies have used government statistics at a regional or national level to quantitatively assess how soybean unit-area yield changes with land size and production inputs. Based on current measurements and assessments, this study provides useful information for understanding and improving soybean productivity in Japan and other Asian countries that encounter similar problems.

## 2 Materials and method

### 2.1 Data preparation

The 1987–2015 soybean production cost statistics reported by the Ministry of Agriculture, Forestry, and Fisheries (MAFF), Government of Japan (a total of 29 years) [23] were used, similar to Kobayashi and Kunimitsu [15], who measured the Törnqvist TFP. The source data for the statistics were collected by the MAFF from representative soybean farms selected nationwide based on the pentennial National Census of Agriculture and Forestry, which ranged between 260 and 490 farms depending on the year. The data were compiled for six sections of land-size ranges, i.e., <0.5 ha, 0.5–1.0 ha, 1.0–2.0 ha, 2.0–3.0 ha, >3.0 ha, and >5.0 ha, in each of the following two agroclimatic regions comprising the land of Japan: (i) the northern subarctic Hokkaido island with large upland farming (hereafter referred to as the Hokkaido region) and (ii) the southern temperate islands, including the main island of Japan, with small paddy farming and rainy season (hereafter referred to as the main-island region). The agricultural land area per farm and proportion of full–time farmers in the Hokkaido region were 28.9 ha and 70 %, respectively, in 2015, whereas those in the main-island region was 2.2 ha and 32 %, respectively. The main-island region accounted for an average of 85 % of the total soybean cultivation area in Japan between 1987 and 2015. Regional mean data of land-size sections were also available in the statistics with the country mean.

The nominal expense values of 12 production-related input elements used for soybean production (i.e., seed, fertilizer, agrochemicals [pest, insect, and herb sides], power, land improvement including irrigation, tax, miscellaneous materials, production management, warehouse, machinery, vehicles, and rental service) were divided by the relevant price indexes of agricultural materials [33] as deflators to calculate the real expense values per farm as their input quantities for each land-size section in the two regions. The real expense values calculated for the first eight input elements were aggregated and regarded as the input quantity of ordinary goods of variable cost, whereas those of the latter four input elements were regarded as the input quantity of mechanical equipment of fixed cost. The quantities for labor and land inputs were the farmers’ working hours and land area for soybean production per farm, respectively. In addition, the aggregate output was the total soybean yield per farm. The same real–value calculations and aggregations were conducted for the regional and country mean data. These data on a fer-farm basis were prepared and used to measure the TFP using the Färe–Primont index. Consequently, two panel datasets with a time series of 29 years were prepared: one consisting of the regional mean values, and the other consisting of the cross–sectional values for the two regions, hereafter referred to as the regional and cross–sectional data, respectively.

The real expense values (i.e., quantities) of the various input elements per unit area were also prepared as explanatory variables for regression analyses after TFP measurement. For the regression analysis of TFP, it was ideal; however, it was difficult to prepare the explanatory variables representing concrete management practices from land preparation to harvest (e.g., variety, type of fertilizer, and sowing method) in the long term across the country. Therefore, this study used the per-unit-area quantitative input values resulting from these practices, while confirming the absence of the potential endogeneity problem from their partial involvement in calculating the aggregate input quantity per farm for the TFP measurement. According to O'Donnell [27,28] who reported the Färe–Primont TFP, the difference in TFP between observations is partly caused by changes in the input mix (composition) and economies of scale (aggregate input quantity). Therefore, although TFP is calculated by controlling for the quantity of aggregate inputs, it increases or decreases with changes in the input mix and different returns to scale between observations. A study by Sheng et al. [34] in Australia showed that TFP improved from small to large farms by adopting different input mixes. Therefore, the use of the prepared quantitative input variables was considered warranted for regression analyses to search for the input elements involved in the input mix changes and economies of scale that affect soybean TFP and yield.

### 2.2 Measurement of TFP by DEA

According to O'Donnell [28] and Anik et al. [29], the TFP of the *n*'s decision–making unit at time *t* (DMU_*nt*_) is expressed as the ratio of its aggregate production output (Q_*nt*_) to its production input (X_*nt*_) as follows:

(1) $${TFP}_{nt=}\frac{Q_{nt}}{X_{nt}}$$

Therefore, the ratio of TFP change in DMU_*nt*_ relative to the *m*'s DMU at time *s* (DMU_*ms*_) is expressed as

(2) $${TFP}_{ms,nt}=\frac{{TFP}_{nt}}{{TFP}_{ms}}=\frac{Q_{nt}/X_{nt}}{Q_{ms}/X_{ms}}$$

When Q_*nt*_ and X_*nt*_ are the distance function D_O_ (x_*0*_, q_*nt*_, t_*0*_) and D_I_ (x_*nt*_, q_*0*_, t_*0*_), respectively, on the reference entity “0” in the DEA by linear programing, Equation (2) is also expressed as:

(3) $${TFP}_{ms,nt}=\frac{D_{O}\left(x_{0},{\;q}_{nt}{,t}_{0}\right)}{D_{O}\left(x_{0},{\;q}_{ms}{,t}_{0}\right)}\frac{D_{I}\left(x_{ms},{\;q}_{0}{,t}_{0}\right)}{D_{I}\left(x_{nt},{\;q}_{0}{,t}_{0}\right)}$$

where the right-hand side is the ratio of the TFP change derived from the Färe–Primont index; and *x* and *q* denote the vectors of production inputs and outputs, respectively. For this study, the production inputs consisted of the per-farm input quantities of ordinary goods, mechanical equipment, labor, and land. The outputs were the total soybean yield per farm, and the DMU was the agroclimatic region or land-size section. The DEA results were reported to be sensitive to the presence of extreme values or outlier [30,31]. However, the probability of presence was considered low for this study, as the statistical data used were mean values over representative farms in each region or land-size section, and further aggregated over some input elements before the DEA, as explained in 2.1 section.

The “fareprim” function of the package “productivity” [35] in R (R Core Team) [36] was used to calculate the Färe–Primont TFP changes in Equation (3) under the assumption of “variable returns to scale” and “both input–output orientations”. The package's “Levels” function was also used to obtain the Färe–Primont TFP level value at each observation. The Färe–Primont TFP changes can be decomposed into components such as technical changes and scale- and mixed-efficiency changes [27]. However, this study used only the TFP level value for regression analyses, as the preliminary analysis confirmed the reliability of the value by comparing it with the Törnqvist TFP.

### 2.3 Regression analysis

The annual change rates of regional TFP and yield were estimated using regional data and the following least-squares linear regression model:

(4) $$\ln\left(Y_{i,t}\right)=\alpha_{i}+\rho_{i}{\text{Year}}_{i,t}+e_{i,t}$$

where Y is the Färe–Primont TFP or unit-area yield in logarithmic form; Year is the annual trend variable, set to 1 for 1987; and *i* and *t* denote the regional identity and the trend variable, respectively. The “lm” function of the R package “stats” was used to estimate the coefficient ρ_*i*_ presenting the annual change rate in the *i–*th region.

Furthermore, the effects of land size, year progress, and production-related input elements on TFP and yield were evaluated using cross–sectional data from each region in the following linear regression models:

(5) $$\ln\left(Y_{j,t}\right)=\alpha +ɤ\;\ln\left({\text{Size}}_{j,t}\right)+\rho {\text{Year}}_{j,t}+\mu_{j}+e_{j,t}$$

(6) $$\ln\left(Y_{j,t}\right)=\alpha +\beta_{k}\;\ln\left({\text{Input}}_{k,j,t}\right)+ɤ\;\ln\left({\text{Size}}_{j,t}\right)+\rho {\text{Year}}_{j,t}+\mu_{j}+e_{j,t}$$

where Size is the land size (i.e., soybean–cultivating land area per soybean–producing farm) in logarithmic form; Input is the logarithmic real expense value (i.e., quantity) of individual input element per unit area, or the logarithmic farmer's working hours per unit area as labor input; μ denotes the unobservable individual effect of land-size section; and *k* and *j* represent the input and land-size section identities, respectively. The coefficients ɤ and β_*k*_, referred to as elasticity in economics, display the ratio of the TFP or yield change to the ratio of change in the land size and *k–*th input, respectively. For model estimation, four of the twelve input elements (tax, miscellaneous material, local management, and vehicles) were dropped owing to a high proportion of missing values or a small share of total expenses. Regression model (5) without the Input variables was used to evaluate the effect of land size, including size–associated input changes. The Year effect (coefficient ρ) in this model was regarded to display the annual progress of land-size–independent production and cultivation technologies, since the technology level changes gradually with time as knowledge and technology is accumulated and disseminated. When the Year effect displayed a negative value, the level of land-size–independent technologies were regarded as being retrogressed with time, which is, technological retrogression. The regression model (6) was estimated to assess the effects of individual input elements by first including all the Input variables as a full model and subsequently selecting Input variables using the backward stepwise selection method based on the adjusted *R*^*2*^ value as the selected model.

The “plm” function of the R package “plm” developed for panel data analysis [37] was used for the estimation of regression model (5) and (6), in which the *Lagrange multiplier* test of the individual effect μ_*j*_ with the “plmtest” function and the Wu–Hausman's specification test for panel models with the “phtest” function were performed to choose the estimation method from the ordinary least–squires, random–effect, and fixed–effect estimations [38]. For the model estimation, the <0.5 ha land-size section in the Hokkaido region and the labor input in the main-island region were dropped owing to a high proportion of missing values and high collinearity with other explanatory variables over the variance inflation factor threshold of 10, respectively. The Wu–Hausman test of endogeneity was conducted using explanatory variables in previous year (i.e., Input_*k,j,t-1*_ and Size_*j,t-1*_) as instrument valuable with the R package “ivreg”: as the result, the existence of endogeneity bias was not detected for all the models analyzed. The stationarity of the explained variables was confirmed by the tests available in the “purtest” function of the “plm” package. Significance tests were performed on the robust standard errors adjusted for heterogeneity and serial correlation of variance observed, as per the instructions in the package manual. The effects of the explanatory variables with statistical significance at the <10 % probability level were focused on. This threshold was adopted considering both the use of non–experimental government statistics and the primary stage of searching for input elements associated with TFP, and yield based on the statistics.

## 3 Results

### 3.1 Trend of TFP and yield

**Fig. 1:**
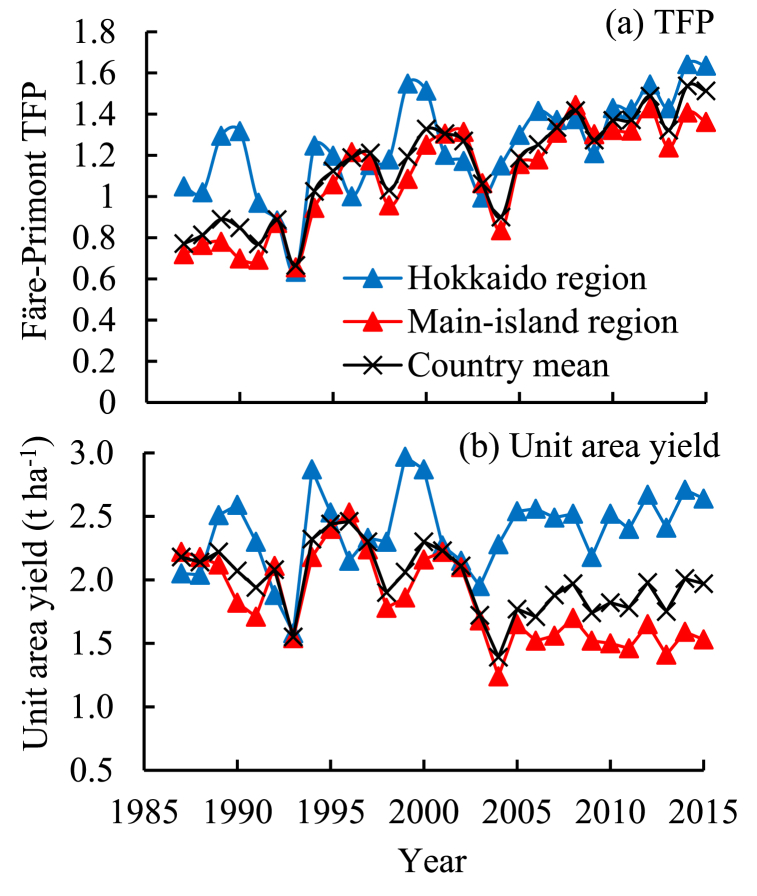
Change in soybean (a) TFP and (b) unit-area yield in the two agroclimatic regions of Japan with the mean country values as reference. TFP, total factor productivity. The TFP was measured as the Färe–Primont index by data envelope analysis using soybean production cost statistics [23]. The yield data were also reported by Kobayashi and Kunimitsu [15].

**Fig. 2:**
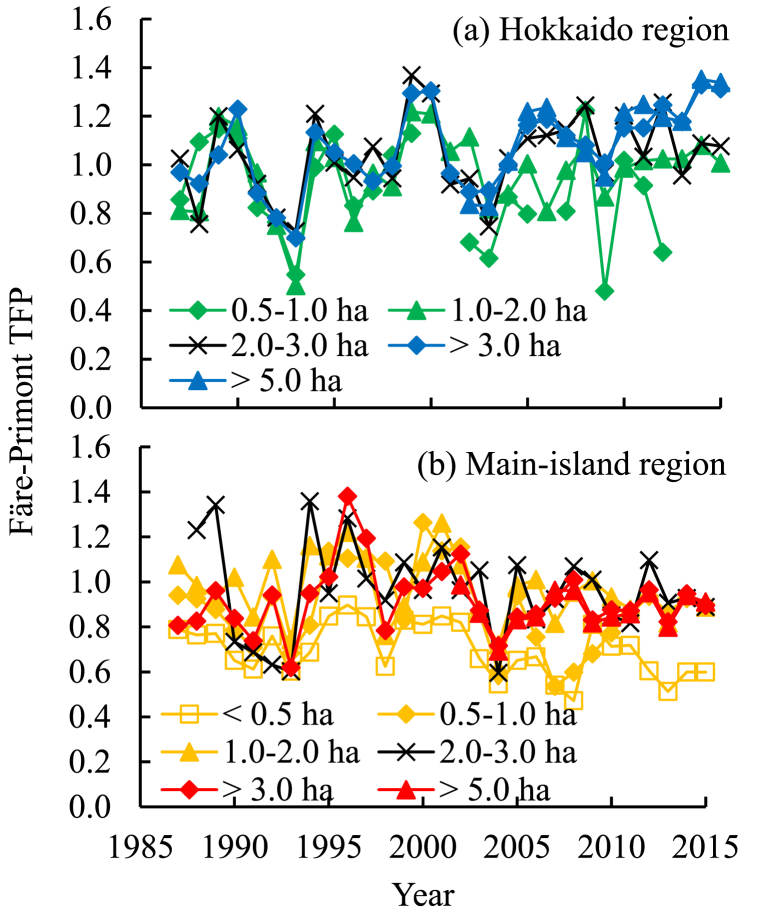
Change in soybean TFP in different land-size sections in the (a) Hokkaido and (b) main-island regions of Japan. TFP, total factor productivity. The land size refers to the soybean–cultivation land area per farm. Overlapping symbols were allowed, since a precise presentation of the inter–sectional differences was not required. Refer to Fig. 1 regarding the TFP measurement.

The Färe–Primont TFP values increased gradually between 1987 and 2015 in both the Hokkaido and main-island regions (Fig. 1). The annual TFP growth rate (ρ value) was estimated at 1.5 % and 2.4 %, respectively, with a 2.3 % annual growth in the country (Table 1). The overall TFP growth during this period was calculated as 45 % and 71 % for the Hokkaido and main-island regions, respectively. The unit-area yield increased by 0.6 % annually for the Hokkaido region; however, it adversely decreased by 1.5 % and 0.7 % for the main-island region and the country, respectively (Fig. 1; Table 1). Gradual decreases in TFP were observed for land-size sections in the main-island region (Fig. 2), in contrast to an increase in regional TFP (Fig. 1).

**Table 1:** Regression of soybean TFP and unit-area yield against the time–trend variable between 1987 and 2015 in two agroclimatic regions of Japan.

Explained variable – Region	ln (TFP or Yield_*i,t*_) = α_*i*_ + ρ_*i*_Year_*i,t*_^a^ + e_*i,t*_				
	α	ρ^a^	Adjusted *R*^*2*^	*F* statistic	Change induced (%)^b^
Färe–Primont TFP^c^					
Hokkaido	−0.02	0.015∗∗∗	0.68	32.4∗∗∗	45
Main island	−0.30∗∗∗	0.024∗∗∗			71
(Country mean	−0.23∗∗∗	0.023∗∗∗)			
Unit-area yield (t ha^−1^)					
Hokkaido	0.77∗∗∗	0.006∗	0.97	432.2∗∗∗	17
Main island	0.81∗∗∗	−0.015∗∗∗			−42
(Country mean	0.78∗∗∗	−0.007∗∗)			

^#^, ∗, ∗∗, and ∗∗∗ indicate the 10 %, 5 %, 1 %, and 0.1 % levels of statistical significance. TFP, total factor productivity. Refer to Fig. 1 for the TFP measurement.

a The Year is the annual trend variable, set at 1 for the year 1987.

b Percent change of TFP or yield induced by the explanatory variable change during the 1987–2015 period as calculated on the regression coefficient.

### 3.2 Effects of the land-size and time progress

The regression coefficients of model (5) for the Hokkaido region in Table 2 displays that the TFP increased significantly by 8.5 % and 0.5 % by doubling of the land size and annual time progress, respectively. Similarly, the yield in the region increased with time however not with land size. Using the regression coefficients estimated that the land-size increase and time progress during the measurement period raised the TFP by 9 % and 15 %, respectively, and the latter progress increased the yield by 15 % (Table 2).

**Table 2:** Regression of soybean TFP and unit-area yield against the land-size and time–trend variables for the 1987–2015 period in the two agroclimatic regions of Japan.

Region – Explanatory variables	Unit	Ratio of change during the period^a^	ln (TFP or Yield_*j,t*_) = α + ɤ ln (Size_*j,t*_) + ρYear_*j,t*_ + μ_*j*_ + e_*j,t*_						
			Färe–Primont TFP				Yield (t ha^−1^)		
			Coefficient		Change induced (%)^b^		Coefficient		Change induced (%)^b^
Hokkaido regionrowhead									
Land size	ha farm^−1^	1.0	0.085∗∗∗	9		−0.011		–	
Year	year	29.0	0.005∗∗	15		0.005∗∗		15	
Estimation method^c^			OLS			OLS			
Number of observations^d^			119			119			
Adjusted *R*^*2*^			0.20			0.05			
*F* statistic			15.96∗∗∗			3.80∗			
Wu–Hausman statistic for the endogeneity			0.38			0.34			
Main-island regionrowhead									
Land size	ha farm^−1^	2.4	0.059^#^	14		−0.039∗		−9	
Year	year	29.0	−0.004∗	−11		−0.013∗∗∗		−37	
Estimation method^c^			RE			RE			
Number of observations^d^			158			158			
Adjusted *R*^*2*^			0.03			0.29			
*χ*^2^ statistic			6.96∗			73.51∗∗∗			
Wu–Hausman statistic for the endogeneity			<0.01			0.08			

The land size refers to the soybean-cultivation land area per farm. OLS, ordinary least–squares estimation; RE, random–effect estimation; TFP, total factor productivity. Refer to Fig. 1 regarding the TFP measurement and to Table 1 for the superscripts indicating statistical significance.

a The ratio was calculated as the 2015 value divided by the 1987 value in logarithmic form, except for the Year variable which ratio was calculated in normal form.

b Percent change of TFP or yield induced by the explanatory variable during the period as calculated on the regression coefficient.

c The method was chosen by the *Lagrange multiplier* test of individual effect μ_*j*_ and the Hausman specification test.

d The number of observations after dropping those with missing values.

For the main-island region, the TFP increased significantly by 5.9 % by doubling the land size, while it decreased by 0.4 % as annual time progressed (Table 2). The yield in the region decreased by 3.9 % and 1.3 % by the land-size doubling and time progress, respectively. Thus, both the TFP and yield in this region decreased significantly with time, in contrast to those in the Hokkaido region. Using the regression coefficients, the land-size increase and time progress during the period was estimated to increase and decrease the TFP by 14 % and 11 %, respectively, with a decrease in yield of 9 % and 37 %, respectively (Table 2).

### 3.3 Other factors affecting the TFP and yield

The regression coefficients in model (6) for the Hokkaido region in Table 3 estimated that the TFP increased by 7–12 % owing to the reduction in fertilizer input and increased use of rental services during the measurement period. Furthermore, the yield in this region improved by 1–11 % by increasing the inputs of seed, agrochemicals, and rental services, in addition to reducing fertilizer input, and it declined by 2–15 % by reducing warehouse and labor inputs.

**Table 3:** Regression of soybean TFP and unit-area yield against the land-size, time–trend, and production-related input variables for the 1987–2015 period in the two agroclimatic regions of Japan.

Region – Explanatory variables	Unit^c^	Ratio of change during the period^a^	ln (TFP or Yield_*j,t*_) = α + β_*k*_ ln (Input_*k,j,t*_) + ɤ ln (Size_*j,t*_) + ρYear_*j,t*_ + μ_*j*_ + e_*j,t*_					
			Färe–Primont TFP			Yield (t ha^−1^)		
			Full model	Selected model^c^	Change induced (%)^b^	Full model	Selected model^c^	Change induced (%)^b^
Hokkaido region								
Seed	REV ha^−1^	0.09	0.07	–	–	0.11^#^	–	1
Fertilizer	ditto	−0.24	−0.27∗∗∗	−0.27∗∗∗	7	−0.11∗	−0.11∗	3
Agrochemicals	ditto	0.35	0.10	0.11	–	0.15	0.17^#^	6
Power	ditto	0.51	−0.18	−0.15	–	0.01	–	–
Land improvement	ditto	−1.13	−0.03	−0.03	–	<0.00	–	–
Rental service	ditto	1.55	0.08∗∗∗	0.08∗	12	0.08∗∗	0.07∗∗∗	11
Warehouse	ditto	−0.32	0.08∗	0.08∗	−3	0.08∗∗	0.07∗∗	−2
Machinery	ditto	−0.06	0.01	–	–	<0.00	–	–
Labor	hr ha^−1^	−0.67	0.04	–	–	0.22^#^	0.22∗∗	−15
Land size	ha farm^−1^	1.04	0.10∗∗∗	0.09∗∗∗	10	0.11∗∗	0.11∗∗∗	11
Year	year	29	−0.001	−0.001	–	−0.001	>0.000	–
Estimation method^a^			OLS	OLS		OLS	OLS	
Number of observations^a^			118	118		118	119	
Adjusted *R*^*2*^			0.28	0.30		0.09	0.11	
*F* statistic			5.17∗∗∗	7.90∗∗∗		2.00∗	3.04∗∗	
Wu–Hausman statistic for the endogeneity			1.26	0.72		1.38	1.03	

Region – Explanatory variables	Unit	Ratio of change during the period^a^	ln (TFP or Yield_*j,t*_) = α + β_*k*_ ln (Input_*k,j,t*_) + ɤ ln (Size_*j,t*_) + ρYear_*j,t*_ + μ_*j*_ + e_*j,t*_					
			Färe–Primont TFP			Yield (t ha^−1^)		
			Full model	Selected model^c^	Change induced (%)^b^	Full model	Selected model^c^	Change induced (%)^b^
Main-island region								
Seed	REV ha^−1^	−0.01	−0.07	–	–	0.01	–	–
Fertilizer	ditto	−0.73	−0.09∗	−0.10∗	7	0.06^#^	0.06∗	−4
Agrochemicals	ditto	0.28	0.09	–	–	0.17^#^	0.16^#^	5
Power	ditto	−0.63	−0.27∗∗	−0.27∗∗∗	17	−0.16∗	−0.17∗∗	10
Land improvement	ditto	−1.10	−0.10^#^	−0.10^#^	11	−0.01	–	–
Rental service	ditto	0.61	<0.00	–	–	0.01	–	–
Warehouse	ditto	−0.43	−0.07	−0.08^#^	3	−0.08∗	−0.08∗∗	4
Machinery	ditto	−0.12	0.10^#^	0.12^#^	−1	0.10	0.09	–
Labor	hr ha^−1^	−1.68	–	–	–	–	–	–
Land size	ha farm^−1^	2.41	<0.00	−0.01		−0.06∗	−0.06∗∗∗	−14
Year	year	29	−0.010∗∗∗	−0.009∗∗∗	−28	−0.015∗∗∗	−0.014∗∗∗	−41
Estimation method^a^			RE	RE		OLS	OLS	
Number of observations^a^			158	158		158	158	
Adjusted *R*^*2*^			0.17	0.18		0.38	0.39	
*χ*^2^ statistic for RE; *F* statistic for OLS			41.55∗∗∗	41.01∗∗∗		10.73∗∗∗	15.57∗∗∗	
Wu–Hausman statistic for the endogeneity			1.15	0.90		0.85	0.71	

a REV, real expense value; TFP, total factor productivity. The land size refers to the soybean-cultivation land area per farm. Refer to Fig. 1 regarding the TFP measurement, to Table 1 for the superscripts indicating statistical significance, and to Table 2 for the superscripts.

b Percentage of TFP or yield change induced by the explanatory variable during the period, as averaged over the two models.

c The explanatory variables were selected by the backward stepwise selection method based on the adjusted *R*^*2*^ value.

For the main-island region, the TFP was estimated to improve by 3–17 % owing to the input reduction of fertilizer, power, land improvement, and warehouses. It decreased by only 1 %, owing to the reduction in machinery (Table 3). Furthermore, the yield in this region improved by 4–10 % from increasing agrochemical input together with reducing the inputs of power and warehouses, and it declined by 4 % from reducing the input of fertilizer.

## 4 Discussion

The Färe–Primont soybean TFP increased at 1.5–2.4 % annually in the two agroclimatic regions between 1987 and 2015, despite an annual decrease of 0.7 % in Japan's average yield (Fig. 1; Table 1). Although these TFP growth rates were slightly larger than those (0.7–1.0 %) measured as the Törnqvist soybean TFP for the two regions by Kobayashi and Kunimitsu [14,15], the previous and current results demonstrate that soybean TFP continually improved across Japan despite a gradual decline in the yield. Similar annual TFP growth rates have been reported with stagnant yields for soybean production in China and India, which use non–GMO cultivars, as in Japan (Appendix 1; [16,17,25]), and likewise for rice production in Japan, where soybean fields are often cultivated by rice farmers or in rotation with rice [30,31]. These past and current results further indicate that there are common factors involved in TFP and yield changes in soybean production in the three Asian major producing countries and between rice and soybean production in Japan. Thus, research collaboration between countries and crops is essential to clarify and understand the common factors affecting soybean TFP and yield.

The regression analyses for the subarctic Hokkaido region estimated that the TFP improved by 9 % and 15 % due to the land-size increase and progress of time, respectively, from the 1987–2015 period (Table 2). For the other temperate main-island region, the TFP was also improved by 14 % due to the land-size increase; however, the improvement was largely offset by the time progress effect (−11 %). These results are the first to clarify that land size increases are a common factor in improving soybean TFP across Japan. This also emphasizes the importance of government policies and technology development for increasing land size, although the negative land size effect on yield in the main-island region requires attention as explained later. Sheng et al. [34] estimated the Cobb–Douglas production function in Australian broadacre farms and found that productivity improved from small to large farms by increasing returns to scale (economies of scale) and changing the input mix (production technologies). Therefore, an efficient improvement in soybean TFP by increasing land size requires further understanding of the input mix change along with the increase and the effect of the change on TFP, as is also discussed later.

The results above further illustrate that the 15 % TFP growth occurred irrespective of the land-size increase in the subarctic Hokkaido region. This means that soybean farmers in this region advanced the level of land-size–irrelevant production technologies (e.g. by using new cultivars and optimizing sowing date). A similar extent of land-size–irrelevant growth (increase) was observed in the yield in this region (Table 2), suggesting that technological progress occurred, with respect to cultivation methods. The local government of Hokkaido has sustained excellent agricultural research and extension organizations [39], likely due to the large-scale farming and the high proportion of full–time farmers explained in section 2.1. In addition, the increase in land size in Hokkaido was slower during the measurement period relative to that in the main-island region (3 vs. 12 times; Appendix 2). Such conditions could motivate soybean farmers in Hokkaido to adopt and improve land-size–irrelevant cultivation technologies developed and recommended by local research and extension organizations. Adversely to the Hokkaido region, a 11 % TFP decline occurred with a 37 % yield reduction irrelevantly to the land size in the temperate main-island region, which accounted for 85 % of the soybean cultivation area in Japan (Table 2). This means that it has been difficult for most soybean farmers in Japan to sustain the same level of land-size–irrelevant cultivation technologies (e.g., sowing date and manure application) as in the past, thereby resulting in technological retrogression. One possible cause for the result is the lack of labor or time for cultivation management owing to the aging of farmers. Another likely cause is decreased soil fertility in soybean fields. Nishida [7] reviewed past analyses of soybean fields converted from paddy fields in the main-island region and concluded that soil nitrogen fertility in the fields decreased gradually with soybean cultivation and was responsible for the low yield. This review indicates that the gradual decrease in soil fertility has reduced soybean yield in this region and, consequently, TFP. Collectively, the current regression analyses that control for land size in each region clarified the inter–regionally different progress in land-size–irrelevant cultivation technologies in Japan and showed that further studies of the causes for the regional differences are required to improve the level of technology across Japan.

Past studies using statistical data in Japan displayed 7–13 % decreases in yield for land area sections above 3–5 ha in rice, sugar beet, and soybean production as compared with smaller land sections ([15,23,40,41]; Appendix 4). Considering these results and the large proportion of soybean cultivation area (85 %) explained by the main-island region, the 12-fold land-size expansion in this region was assumed to be one of the factors decreasing soybean yield in Japan (Appendix 2; Fig. 1). The current regression analyses estimated that a 9 % decrease in the yield in the region was attributable to the land-size increase, thereby confirming the land-size effect on the yield (Table 2). This result also implies that the TFP–improving land-size increase leads to an adverse decrease in yield in the region and that scientists need to consider land size in soybean yield analyses across different farms and years. Large soybean farms in the region tend to reduce fertilizer input, which displayed a positive regression coefficient for yield (Appendix 4; Table 3). Thus, this input reduction could underlie the observed decrease in yield with increasing land size. These results further imply for policy makers that the measures and subsidies required for soybean farmers to increase the land size could result in reducing the yield, and that sustaining the yield level while increasing the land size requires attention and countermeasures against input elements to be reduced with the increase. However, it should be noted that relative to the land size effect, the time–progress effect induced a much greater yield decrease (37 %) in the region (Table 2), thereby showing that the primary cause of the yield decrease in Japan was the retrogression of cultivation technologies, which is explained in the previous paragraph.

The TFP improved by 38 % collectively owing to the input reduction of fertilizer, power, land improvement, and warehousing in the main-island region, and by 7 % due to the reduction of fertilizer in the Hokkaido region (Table 3). Thus, saving production inputs per unit area, especially fertilizers, is a major option for soybean farmers in Japan to improve their TFP. For the main-island region, involving these input elements in regression model (6) eliminated the positive land-size effect that explained the 14 % TFP increase in model (5) without these elements (Table 2, Table 3). This suggests that the land-size effect observed on TFP for this region occurred via the above–mentioned input reduction and that such a reduction in particular elements possibly induce TFP–improving input mix change by the land-size increase [27,28,34].

Previous studies from Asia and the United States of America reported the positive effects of irrigation on soybean TFP and yield [[17], [18], [19],^25^]. However, the current study measured negative regression coefficients in the land improvement input, including irrigation, against the TFP for the main-island region. In addition, reducing the input quantity was found to improve the TFP by 11 % without sacrificing the yield. This may imply that for policymakers, the quantity of land improvement input at the farm level was not a limiting factor for raising soybean TFP and yield in Japan and was excessive in terms of TFP. A positive effect of mechanization on soybean TFP was reported in China [17]. Similarly, this study detected a positive effect (regression coefficient) of machinery input on TFP in the main-island region. However, the machinery input in this region was reduced by 12 % during the measurement period, thereby resulting in a 1 % TFP decrease (Table 3). This result further implies that for policymakers and machinery scientists, the positive effect of machinery input was not sufficient to improve TFP, further requiring measures for soybean farmers to increase machinery input for the improvement.

Increases in agrochemical input during the period improved the yield by 5–6% in the two regions without reducing the TFP (Table 3). The yield decreases owing to soil–borne diseases and weed problems resulting from continuous cropping and excessive field water in fields converted from paddy fields [5,9]. Perhaps, such biological field problems were mitigated by an increase in agrochemical input. Positive agrochemical effects on soybean yield were reported in past yield analyses across different states in the United States of America, supporting the potential of this input element to increase the yield [19,21,22]. Similar to agrochemical input, the input elements whose quantity per unit area increased to improve yield or TFP without sacrificing the other will be promising targets for future research and development, since production technologies using such input elements are more likely to be adopted by farmers than those using decreased input elements. Seed and rental services were also identified as such input elements for the Hokkaido region, together with land size, which increased to improve TFP (Table 3). The current regressing analyses that led to the above findings used only production-related input elements as explanatory variables, thus not considering external environmental and economic factors that can affect TFP and yield (e.g., air temperature, flood damage, soybean price, agricultural infrastructure). Further regression analyses that produce and involve explanatory variables presenting such external factors are necessary for not only clarifying their effects on TFP and yield, but also precisely estimating the effect of each input element.

## 5 Conclusions

This study measured Färe–Primont soybean TFP over 29 years in two agroclimatic regions in Japan to assess the factors determining TFP and yield changes in consideration of land size. Our results illustrate (i) continual TFP improvements across Japan, despite a gradual decrease in yield; (ii) increasing land size and reducing production inputs as common TFP–improving factors in the two regions; and (iii) the progress and retrogression of production or cultivation technologies depending on the region. However, the TFP–improving land size increase resulted in a decrease in yield in the main-island region accounting for 85 % of soybean cultivation area in Japan. Regression analyses also identified some input elements that could improve the TFP or yield without sacrificing the other. The implications of the results of this study are discussed for policymakers and scientists from the perspective of improving both TFP and yield in Japan while increasing land size. Further studies that involve more production-related explanatory variables in the regressions or farm–level production cost data are required to clarify the factors responsible for the technological progress and retrogression observed and to determine further concrete targets and robust strategies for TFP and yield improvements.

## CRediT authorship contribution statement

**Sohei Kobayashi:** Writing – original draft, Visualization, Project administration, Methodology, Funding acquisition, Formal analysis, Data curation, Conceptualization. **Yoji Kunimitsu:** Writing – review & editing, Methodology.

## Data availability statement

All data used for this study are publicly available as explained in this article.

## Funding

This study was supported by a Grant-in-Aid for Scientific Research from the 10.13039/501100001691Japan Society for the Promotion of Science [grant number JP20K06269] to S. Kobayashi and Y. Kunimitsu.

## Declaration of competing interest

The authors declare that they have no known competing financial interests or personal relationships that could have appeared to influence the work reported in this paper.

## Figures and Tables

**Appendix 1:**
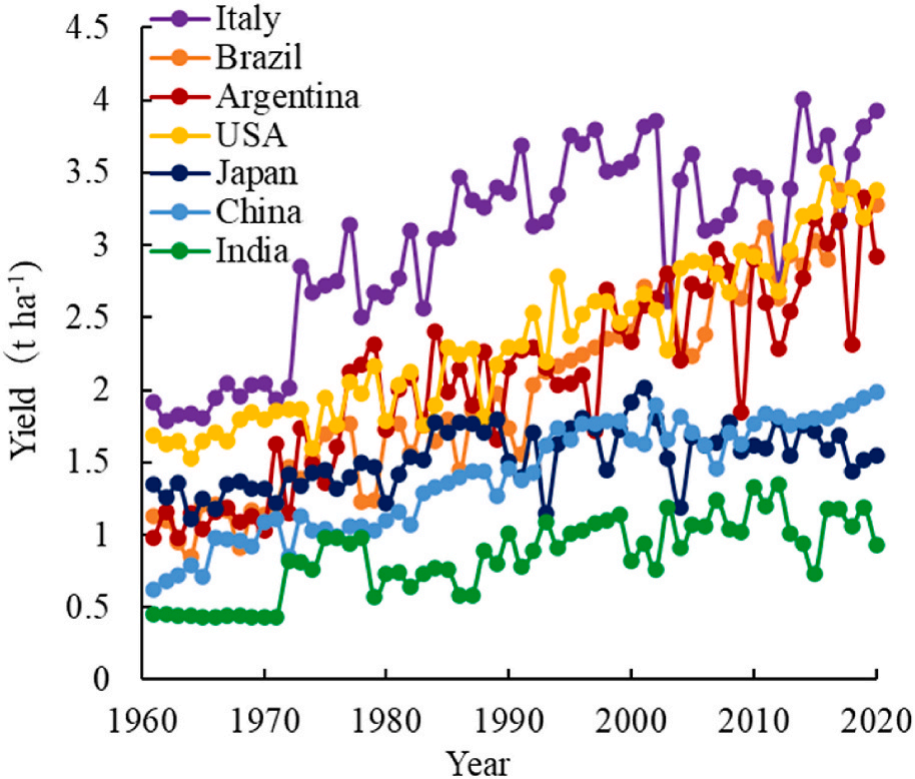
Change in soybean yield in major production countries. The data were retrieved from the FAO [1].

**Appendix 2:** Soybean–producing land area per soybean–producing farm in Japan.

Year	Per-farm land area (ha)		
	Country	Hokkaido	Main island
1987	0.31	1.41	0.26
2015	3.40	4.06	3.05
Ratio of change	10.8	2.9	11.6

The data were retrieved from soybean production cost statistics [23].

**Appendix 3:** Soybean–producing land area per employee in the agriculture sector in three soybean–producing countries in Asia.

Year	Per-employee land area (ha)		
	Japan	China	India
1991	0.032	0.018	0.016
2020	0.061	0.051	0.063
Ratio of change	1.9	2.8	4.0

The values were calculated by the following formula: soybean area harvested/(total labor force ･ percent employment in agriculture / 100). The data of the area harvested and the other two were retrieved from the FAO [1] and World Bank [24], respectively.

**Appendix 4:** Indexes of per-unit-area input quantity and soybean yield by different land-size sections in the main-island region of Japan.

Input and output	Section of land size (ha)			
	0.5–1.0	1.0–2.0	2.0–3.0	>3.0
Seed	100	98	98	96
Fertilizer	100	91	87	69
Agrochemicals	100	100	98	96
Power	100	95	88	94
Land improvement	100	91	74	39
Rental service	100	104	91	48
Warehouse	100	81	77	74
Machinery	100	104	108	141
Labor	100	69	58	48
Yield	100	108	104	94

The land size refers to the soybean-cultivation land area per farm. The indexes of each input and yield were calculated by the following formula: the real expense value or yield averaged from 2006 to 2015 / the 0.5–1.0 ha section value ･ 100. The data were retrieved from soybean production cost statistics [23].
